# Current Treatment Options in Vestibular Migraine

**DOI:** 10.3389/fneur.2014.00257

**Published:** 2014-12-04

**Authors:** Mark Obermann, Michael Strupp

**Affiliations:** ^1^Department of Neurology, Vertigo and Dizziness Center, University of Duisburg-Essen, Essen, Germany; ^2^Department of Neurology, German Center for Vertigo and Balance Disorder, Ludwig-Maximillians-University Munich, Munich, Germany

**Keywords:** vestibular migraine, migraine with aura, migraine, vertigo, dizziness, gait disturbance

## Abstract

Approximately 1% of the general population in western industrialized countries suffers from vestibular migraine. However, it remains widely unknown and often under diagnosed despite the recently published diagnostic criteria for vestibular migraine. Treatment trials that specialize on vestibular migraine are scarce and systematic randomized controlled clinical trials are now only emerging. This review summarizes the knowledge on the currently available treatment options that were tested specifically for vestibular migraine and gives an evidence-based, informed treatment recommendation with all its limitations. To date only two randomized controlled treatment trials provide limited evidence for the use of rizatriptan and zolmitriptan for the treatment of vestibular migraine attacks because of methodological shortcomings. There is an ongoing multicenter randomized placebo-controlled trial testing metoprolol 95 mg vs. placebo (PROVEMIG-trial). Therefore, the therapeutic recommendations for the prophylactic treatment of vestibular migraine are currently widely based on the guidelines of migraine with and without aura as well as expert opinion.

## Introduction

Dizziness and headache are frequently reported symptoms in clinical neurology (1, 2). About 30–50% of all patients with migraine describe vertigo or dizziness associated with their migraine at least occasionally (3, 4). The International Headache Society (IHS), the Bárány-Society (International Society for Neuro-Otology), otorhinolaryngologists, and other scientists have created a consensus document with consistent diagnostic criteria for vestibular migraine (5, 6) that was considered in the appendix of the new ICHD-3 beta version of the international headache classification (7). These internationally accepted diagnostic criteria are intended to further promote the systematic research on vestibular migraine including clinical trials. Furthermore, they serve to create more certainty in diagnostic assessment and clinical management of these patients in everyday clinical practice. It remains an ongoing discussion whether vestibular migraine is an autonomous disorder with distinct pathophysiological features or whether it simply represents a subform of migraine that also includes vestibular symptoms (8).

Approximately two-thirds of the patients experience vertigo or dizziness seek professional medical care because of their symptoms, but only in 20% of all patients vestibular migraine was diagnosed (9). The various interpretations by different specialties regarding the combination of both symptoms, i.e., headache and vertigo, are alarming. A survey among neurologists and otologists reports that neurologists would diagnose a vestibular migraine in 82% of patients with vertigo and headache, while this opinion was shared only by 64.5% of ENT physicians. Surprisingly, in the context of this survey 19% of ENT physicians and even 14.5% of neurologists declared never to have treated a patient with vestibular migraine before (9).

Treatment trials that specifically address vestibular migraine are scarce and most treatment suggestions for vestibular migraine are extrapolated from knowledge gathered from clinical trials on migraine with and without aura. This review summarizes the current knowledge about treatment options that are specific for vestibular migraine.

## Methods

A Medline search was conducted to collect all available published data on the treatment of vestibular migraine. The search terms vestibular AND migraine returned 427 publications including 115 review articles and 18 labeled as clinical trial (October 2014). The combination of “vestibular migraine” returned 115 publications including two labeled as clinical trials and 37 as review articles. Overall, 22 publications were related to the specific treatment of vestibular migraine, not considering the numerous reviews about vestibular migraine and not considering the evidence rating. Some patients enrolled in these trials did not fulfill the later defined diagnostic criteria for vestibular migraine and in some, a different terminology was used. The most common terms to describe the combination of both (migraine and vestibular symptoms) besides vestibular migraine (10) were migrainous vertigo, migraine-associated vertigo, vertiginous migraine, migraine-associated balance disturbance, and benign paroxysmal vertigo.

### Burden of vestibular migraine

In population-based studies the lifetime prevalence of migraine and vertigo in the general population of Germany as a representative of the western industrial nations was approximately 16 and 7%, respectively (1, 11). In the general population, the lifetime prevalence of vestibular migraine was 1%, with a one-year prevalence of 0.9% (12). A large epidemiological study showed the concurrent presence of both symptoms in 3.2% of all patients (1). A different survey described the presence of dizziness or vertigo in 47.5% of patients with migraine during a severe migraine attack with pain intensity of 7 or more on a visual analog scale (VAS 0 = no pain, 10 = worst imaginable pain) (13).

Vestibular migraine can occur in any period of life (10, 14, 15). Women are affected more frequently than men with a gender ratio between 1.5 and 5 (10, 15–17). A familial clustering as well as an autosomal-dominant inheritance with a lower penetrance in males was described (18). In most patients, vestibular migraine appears in an advanced period of life and often with a temporal delay to the first onset of migraine (10, 17). The association of vestibular symptoms with migraine with aura is controversial, as some studies found this connection (10, 17, 19–22), while others discovered that patients with migraine without aura have vestibular symptoms more often or at least equally often as migraine with aura patients (10, 16, 19). In elderly patients, especially in postmenopausal women, the typical migraine attacks are sometimes replaced by isolated episodes of vertigo, dizziness, or transient feeling of disequilibrium (23).

### Clinical presentation

In a large population-based survey the most frequent vertigo symptoms were a spontaneous rotatory vertigo in 67% followed by positional vertigo in 24% of patients with vestibular migraine (12). Initial spontaneous rotatory vertigo can turn into a positional or illusion of movement type vertigo with gait disturbances (24, 25). An increased sensitivity to motion, particularly to movements of the head, or fast-moving visual objects are often part of the attack (26, 27).

The duration of attacks vary from few seconds (10% of patients) to some minutes (30% of patients), some hours (30% of patients), and even up to a few days (30% of patients) (10, 14, 16, 19). Only 10–30% of patients report a typical vestibular aura with duration between 5 and 60 min (10, 17). Approximately 30% of all vestibular migraine attacks are not accompanied by headache (14, 16, 17). In these cases, the typical migraine-associated symptoms like phono-/photophobia, osmophobia, nausea and vomiting, aggravation by movement, or the need to rest are the crucial symptoms for diagnosis (28). Gaze-induced nystagmus, saccadic pursuit (especially vertically), central positional nystagmus, and a slight horizontal or vertical spontaneous nystagmus can be observed during or after a migraine attack and might support the diagnosis (10).

### Basilar-type migraine

Basilar-type migraine, in contrast to vestibular migraine, was already defined in the ICHD-2 classification and can be distinguished clearly from vestibular migraine in most cases (28). In the revised ICHD-3 beta classification, it is termed migraine with brainstem aura (7). Less than 10% of patients with vestibular migraine meet the diagnostic criteria for basilar-type migraine (16, 17). By definition, basilar-type migraine requires at least two aura symptoms, which are assignable to the vertebrobasilar territory, which last between 5 and 60 min, and which are followed by a typical migraine headache (28). The extent to which both kinds of migraine can be distinguished and to what degree there are overlaps in some patients or disease constellations remains subject of current research.

### Medical therapy

There are two randomized controlled clinical studies on the specific treatment of vestibular migraine with triptans (29, 30). This study suggested a benefit from zolmitriptan 5 mg in 38% (three of eight episodes) of patients with vestibular migraine attacks, whereas in the placebo group a positive effect was observed in only 22% (two of nine episodes). Unfortunately, the validity of this study is limited due to the large confidence intervals and the small number of patients recruited (*n* = 10) with only 17 reported attacks (29). A double-blind, randomized, placebo-controlled study with rizatriptan vs. placebo measured motion sickness in response to a complex vestibular stimulus. Subjects included 25 migraineurs with or without migraine-related dizziness (23 females) aged 21–45 years (31.0 ± 7.8 years). Results indicated that of the 15 subjects who experienced vestibular-induced motion sickness 13 showed a decrease in motion sickness after rizatriptan compared to placebo (*p* < 0.02). However, this positive effect was not seen after exposure to more provocative vestibular stimulation. It was suggested by the authors that the serotonin agonist, rizatriptan, reduces vestibular-induced motion sickness by influencing serotonergic vestibular-autonomic projections (30).

All other therapeutic approaches are based on case reports, retrospective cohort studies, and open-label trials (Table 1). Many therapeutic recommendations for vestibular migraine are currently based on the guidelines of migraine with and without aura. This is supported by a large retrospective cohort evaluation on 100 patients (median age 47 years, range 21–72 years) that compared vestibular migraine patients with and without prophylactic migraine treatment (31). All patients on prophylactic treatment showed a decrease of duration, intensity, and frequency of episodic vertigo as well as its associated features (*p* < 0.01). Medications used were beta-blocker [49 patients, metoprolol (69%; median dose 150 mg) or propranolol (31%; median dose 160 mg)], valproic acid (6 patients, 8%; median dose 600 mg), topiramate 6 patients (8%; median dose 50 mg), butterbur extract 4 patients (5%; median dose 50 mg), lamotrigine 3 patients (4%; median dose 75 mg), amitriptyline (2 patients; 100 and 75 mg), flunarizine (1 patient: 5 mg), and magnesium (3 patients; median dose 400 mg). The group without prophylactic therapy showed a reduction of vertigo intensity only (31). A different retrospective study with 100 patients also indicated a positive effect of migraine prophylactic medication in patients with vestibular migraine (32). In the third retrospective cohort study, 33 patients with recurrent vertiginous attacks and migraine were evaluated. The assessment of frequency reduction showed complete disappearance in 19 patients (57.6%), over 50% reduction in 8 (24.2%), under 50% reduction in 5 (15.2%), and no reduction in one patient. Propranolol was used by 12 patients, metoprolol by 2, flunarizine by 7, amitriptyline by 2, and clonazepam by 11 patients (33).

**Table 1:** **Treatment options in vestibular migraine**.

Acute treatment	Dosage	Trial (Reference)
Zolmitriptan	2.5 mg oral	Randomized controlled trial (RCT) (29)
Rizatriptan	10 mg oral	RCT, motion sickness (30)
**PROPHYLACTIC TREATMENT**		
Metoprolol	150 mg oral	Retrospective cohort analysis (31)
	100–200 mg oral	Retrospective cohort analysis (33)
Propranolol	160 mg oral	Retrospective cohort analysis (31)
	40–160 mg oral	Retrospective cohort analysis (32, 33)
Valproic acid	600 mg oral	Retrospective cohort analysis (31)
	600 mg oral	Cohort study, vestibulo-ocluar reflex (34)
Topiramate	50 mg oral	Retrospective cohort analysis (31)
	50–100 mg oral	Open-label, chart review (44)
Butterbur extract	50 mg oral	Retrospective cohort analysis (31)
Lamotrigine	75 mg oral	Retrospective cohort analysis (31)
	100 mg oral	Retrospective, open-label (41)
Amitriptyline	100 mg oral	Retrospective cohort analysis (31)
	10 mg oral	Retrospective cohort analysis (33)
Nortriptyline	25–75 mg oral	Open-label, chart review (44)
Flunarizine	5 mg oral	Retrospective cohort analysis (31)
	5–10 mg oral	Retrospective, open-label (33)
	5–10 mg	Open-label, post-marketing (36, 37)
Magnesium	400 mg oral	Retrospective cohort analysis (31)
Clonazepam	0,25–1 mg oral	Retrospective cohort analysis (33)
Cinnarizine	37.5–75 mg oral	Retrospective, open-label (35)
**NON-MEDICAL TREATMENT**		
Vestibular rehabilitation exercises	5 therapy sessions over 9 weeks	Uncontrolled, observational trial (43)
Caffeine cessation	4–6 weeks	Retrospective, observational trial (44)

Drugs intended for acute treatment such as sumatriptan, non-steroidal anti-inflammatory drugs (NSAID), and ergots as well as preventive migraine medications such as beta blockers, tricyclic antidepressants, methysergide, flunarizine, topiramate, and valproate were studied (33). In a different study, sodium valproate did not relief vestibular symptoms but did have a considerable effect on migraine headache in 8 of 12 patients (34). In this study, the horizontal vestibulo-ocular reflex (VOR) was evaluated by sinusoidal harmonic acceleration test at 0.01, 0.02, 0.04, 0.08, and 0.16 Hz using a computerized rotatory chair system. No abnormalities were found for the VOR gain, phase and asymmetry for any frequency. These normal VOR measurements contrasted with the repeated complaints of dizziness, vertigo and unsteadiness reported by seven patients (58%) that remained unchanged by valproate treatment (34).

Cinnarizine was tested in a retrospective, single-center, open-label, investigation of the effects of cinnarizine on vestibular migraine and migraine associated with vertigo (35). The study included 24 subjects with VM (23 women, 1 man) and 16 subjects with basilar type Migraine (12 women, 4 men). The ages of subjects ranged from 18 to 54 years (mean 30 years). The mean frequency of vertigo and also the mean frequency, duration and intensity of migraine headaches per month were reduced significantly after three months of cinnarizine therapy (all *p* < 0.001) (35). This will have to be reconfirmed in a large scale, randomized, controlled clinical trial.

There are two large open-label post-marketing studies investigating flunarizine for the treatment of migraine and the treatment of vertigo (36, 37). In both conditions, flunarizine showed considerable efficacy compared to propranolol for migraine or betahistine for vertigo. Unfortunately, both studies did not look at patients that suffered from migraine and vertigo specifically and thus the effect of flunarizine can be suspected, but remains unproven. One randomized control trial (RCT) was undertaken in a tertiary academic center in order to evaluate the efficacy of flunarizine in 48 patients with vestibular migraine over 12 weeks in comparison to treatment with betahistine 16 mg and vestibular exercises (38). The frequency of vertiginous episodes decreased with flunarizine treatment (*p* = 0.010) and severity of vertigo also improved (*p* = 0.046). Headache frequency and severity was not significantly different between treatment groups. The main side effects were weight gain and somnolence (38). A different retrospective chart study evaluated the effect of flunarizine and propranolol on vestibular migraine patients. Thirty patients with flunarizine showed a 68% responder rate of VM symptoms (*p* < 0.001). For propranolol, 31 patients showed an improvement of symptoms in 73% (*p* < 0.001) (39).

Lamotrigine was reported to be effective in the treatment of migraine auras, isolated auras, and to a lesser extend migraine-associated headaches (40). One retrospective, open-label study investigated the efficacy of 100 mg lamotrigine in 19 patients (6 men, 13 women) with vestibular migraine over 3–4 months. The average vertigo frequency per month was reduced from 18.1 to 5.4, headache frequency dropped from 8.7 to 4.4, but did not reach statistical significance. Therefore, lamotrigine appears to mainly act on vestibular symptoms and only to a lesser extent on headaches (41). Lamotrigine was also effective in three patients with basilar-type migraine over five years (42).

Less conventional drugs such as benzodiazepines, selective serotonin reuptake inhibitors (SSRI), pizotifen, dothiepin, and acetazolamide, as well as behavioral modification such as special diets were reported to have a positive effect on vestibular migraine (33). However, it is difficult to extract a clear therapeutic recommendation for the particular treatment of vestibular migraine from these data. Furthermore, it must be kept in mind that inconsistent definitions of vestibular migraine were used in the context of these studies. As a result, the examined patient groups were quite heterogenous. In the future, this deficit should be eliminated by the new diagnostic criteria, hopefully leading to more comparable, high quality studies.

### Non-medical treatment options

Vestibular rehabilitation exercises were described to be beneficial in patients with vestibular migraine in addition to medical treatment or as stand alone treatment option (43). Thirty-six patients (vestibular migraine = 20, vestibular impairment = 16) with daily vestibular symptoms received a 9 week vestibular rehabilitation program. Each subject attended five therapy appointments at initial, 2, 5, 9, and 6 months. While the vestibular migraine group showed poorer subjective performance at the onset of therapy, both groups benefited equally from rehabilitation. The same degree of improvement was observed in the migraine group regardless of medication regime. This study suggested that vestibular rehabilitation therapy may be effective in vestibular migraine regardless of the used medical prophylactic therapy (43). Even though, this is in line with the very positive effect of physical activity on the reduction of migraine with and without aura, yet again, a controlled study design is required in order to clearly recommend such a therapy approach.

### Behavioral modification

One interesting study investigated the cessation of caffeine for the treatment of vestibular migraine in 34 patients (44). Fourteen percent of patients reported improvement in symptoms upon caffeine cessation. In comparison, the same study showed that topiramate reduced symptoms in 25% of patients and 46% of patients reported a reduction in dizziness after nortriptyline therapy (*p* = 0.007). In total, 75% of VM patients received sufficient benefit from this therapeutic pathway to not progress to other treatments (44).

### Recommendations for treatment

The treatment recommendations for vestibular migraine are currently similar to those for migraine with or without aura because of the lack of specific trials in that field. Some of the following treatment recommendations are based on the authors’ experience.

According to the available literature zolmitriptan 5 mg (tablet, nasal spray, and dissolvable tablet) should be the first choice in the acute vestibular migraine attack. Rizatriptan may also be used and analogous to migraine any other triptan may be just as effective (45). Patients with excessive nausea or vomiting may prefer non-oral applications (i.e., nasal spray, suppositories or subcutaneous injections) or intravenous options such as acetylsalicylacid 1000 mg where available with metoclopramide 10 mg or dimenhydrinate (62.5 mg). Prophylactic medications are not different from treatment of migraine with or without aura and include propranolol 80–240 mg, metoprolol 50–200 mg, bisoprolol 5–10 mg, or flunarizine 5–10 mg per day in patients with over three attacks per month, with very long lasting or disabling attacks, or failure to respond to acute treatment. Topiramate 25–100 mg and valproic acid 500–600 mg may be used as well. Combination of drugs belonging to the same class is probably not reasonable, and the combination of topiramate with propranolol did not show an additional benefit to topiramate alone (46). Patients with frequent migraine attacks on 15 days per month for >3 months and with >8 days with migraine headache per month for more than 3 months should receive topiramate 25–100 mg. A trial of onabotulinumtoxin type A (155 MU), as used in the treatment of chronic migraine, may also be reasonable, but this option is unlikely to improve vestibular symptoms. Patients who predominantly complain of vertigo or dizziness with typical aura duration, but without frequent migraine headache might be successfully treated with lamotrigine 25–100 mg per day (41).

## Conclusion

So far there are no state-of-the-art published RCTs on the prophylactic treatment of vestibular migraine. There is, however, an ongoing multicenter randomized placebo-controlled trial (metoprolol 95 mg per day vs. placebo; the PROVEMIG-trial). Established treatment regimens for migraine with and without aura can be effective in vestibular migraine, but it remains uncertain whether certain or more specific treatment options would be more beneficial for this special patient group. The knowledge derived from these clinical trials may also be useful for the distinction of vestibular migraine from migraine with and without aura, as well as from basilar-type migraine. Therefore, it could help to disentangle whether vestibular symptoms are merely one of the many migraine features or represent a genuine subform of migraine as suggested by current terminology of vestibular migraine.

## Conflict of Interest Statement

Mark Obermann has received scientific support and/or honoraria from Biogen Idec, Novartis, Sanofi-Aventis, Genzyme, Pfizer, Teva and Heel. He received research grants from Allergan, Electrocore, Heel and the German Ministry for Education and Research (BMBF). Michael Strupp is co-Chief Editor of the *Journal of Neurology*, and Specialty Chief Editor of *Frontiers in Neurology* – *Neuro-otology* and Section Editor of *F1000*. He has received speaker’s honoraria from Abbott, Actelion, UCB, GSK, TEVA, Biogen Idec, Pierre-Fabre, Heel, Nordic, Eisai and Hennig Pharma and has received grants from the Federal Ministry of Education and Research (BMBF) and the Deutsche Forschungsgemeinschaft (DFG).
